# P-1426. Serotype Distribution and Vaccine Coverage of Invasive Pneumococcal Disease in Mexico, Brazil, and Argentina, 2016-2023

**DOI:** 10.1093/ofid/ofaf695.1613

**Published:** 2026-01-11

**Authors:** Amanda M Martino, Juan Urrego-Reyes, Mohan Kumar Paluru, Jan Cervenka, Nicole Cossrow

**Affiliations:** Merck & Co., Inc., Rahway, NJ; MSD Colombia, Bogota, Cundinamarca, Colombia; Accenture plc, Bengaluru, Karnataka, India; Merck, Sharp & Dohme, Prague, Kralovehradecky kraj, Czech Republic; Merck & Co, Inc., Kenilworth, NewJersey

## Abstract

**Background:**

Invasive pneumococcal disease (IPD) is a severe infection in which *Streptococcus pneumoniae* bacterium spreads to normally sterile sites in the body, resulting in meningitis (brain and/or spinal cord infection), bacteremia (bloodstream infection), and bacteremic pneumonia (lung infection). Despite availability of pneumococcal conjugate vaccines (PCV) to prevent IPD in Latin America, a significant burden of disease remains in adults. There is a scarcity of literature on the serotype distribution and vaccine coverage of IPD in this region, which this study aims to address.

**Table 1.**
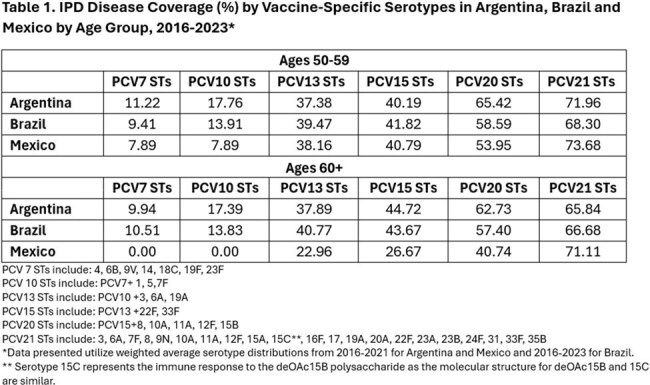
IPD Disease Coverage (%) by Vaccine-Specific Serotypes in Argentina, Brazil, and Mexico by Age Group, 2016-2023

**Table 2.**
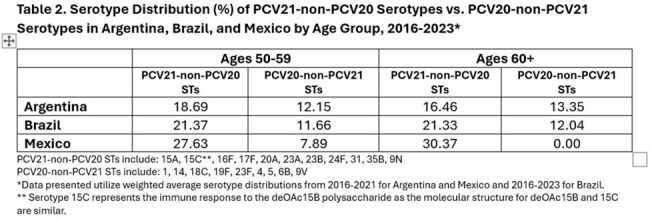
Serotype Distribution (%) of PCV21-non-PCV20 Serotypes vs. PCV20-non-PCV21 Serotypes in Argentina, Brazil, and Mexico by Age Group, 2016-2023

**Methods:**

Publicly available IPD surveillance data from the Pan American Health Organization’s (PAHO) SIREVA II project (Network Surveillance System for the Bacterial Agents Responsible for Pneumonia and Meningitis) from Argentina, Brazil, and Mexico were collated from 2016-2023 (or most recent year of available data per country). Serotype distribution by age group (50-59 years and ≥ 60 years) and vaccine type (PCV7, PCV10, PCV13, PCV15, PCV20, and PCV21) were analyzed over the study period. PCV21 is the new adult-specific 21-valent PCV that was recently approved in US, Canada, Australia, and EU. Disease coverage provided by the unique 11 serotypes in PCV21 not found in PCV20 was summarized for each country by age group.

**Results:**

Serotype distribution of IPD in Latin America primarily includes those contained in PCV21. Disease coverage ranges from 68.30% in Brazil, 71.96% in Argentina, and 73.68% in Mexico for those aged 50-59 years, and 65.84% in Argentina, 66.68% in Brazil, and 71.11% in Mexico for those aged ≥ 60 years (Table 1). The additional population health benefit offered by PCV21 is mostly due to its unique 11 serotypes not found in PCV20, which account for an additional disease coverage ranging from 18.69-27.63% among 50–59-year-olds to 16.46-30.37% among ≥ 60 years olds (Table 2).

**Conclusion:**

Results demonstrate that PCV21 serotypes are associated with a higher number of IPD cases in adults in Argentina, Brazil, and Mexico, compared to serotypes included in other available vaccines. The inclusion of PCV21 in national immunization programs for adults may significantly reduce the residual burden of IPD in this population.

**Disclosures:**

Amanda M. Martino, MPH, Merck & Co., Inc.: Employee|Merck & Co., Inc.: Stocks/Bonds (Public Company) Juan Urrego-Reyes, MD, MSc, MSD Colombia: Employee|MSD Colombia: Stocks/Bonds (Public Company) Mohan Kumar Paluru, n/a, Accenture: Employee|Accenture: Stocks/Bonds (Public Company) Jan Cervenka, n/a, Merck Sharp & Dohme: Employee|Merck Sharp & Dohme: Stocks/Bonds (Public Company) Nicole Cossrow, PhD, Merck & Co., Inc.: Employee|Merck & Co., Inc.: Stocks/Bonds (Public Company)

